# Titration of Long-Acting Insulin Using Continuous Glucose Monitoring and Smart Insulin Pens in Type 1 Diabetes: A Model-Based Carbohydrate-Free Approach

**DOI:** 10.3389/fendo.2021.795895

**Published:** 2022-01-10

**Authors:** Anas El Fathi, Chiara Fabris, Marc D. Breton

**Affiliations:** Center for Diabetes Technology, University of Virginia, Charlottesville, VA, United States

**Keywords:** type 1 diabetes, multiple daily injections, long-acting insulin, insulin titration, continuous glucose monitoring, smart insulin pens

## Abstract

**Objective:**

Multiple daily injections (MDI) therapy is the most common treatment for type 1 diabetes (T1D), consisting of long-acting insulin to cover fasting conditions and rapid-acting insulin to cover meals. Titration of long-acting insulin is needed to achieve satisfactory glycemia but is challenging due to inter-and intra-individual metabolic variability. In this work, a novel titration algorithm for long-acting insulin leveraging continuous glucose monitoring (CGM) and smart insulin pens (SIP) data is proposed.

**Methods:**

The algorithm is based on a glucoregulatory model that describes insulin and meal effects on blood glucose fluctuations. The model is individualized on patient’s data and used to extract the theoretical glucose curve in fasting conditions; the individualization step does not require any carbohydrate records. A cost function is employed to search for the optimal long-acting insulin dose to achieve the desired glycemic target in the fasting state. The algorithm was tested in two virtual studies performed within a validated T1D simulation platform, deploying different levels of metabolic variability (nominal and variance). The performance of the method was compared to that achieved with two published titration algorithms based on self-measured blood glucose (SMBG) records. The sensitivity of the algorithm to carbohydrate records was also analyzed.

**Results:**

The proposed method outperformed SMBG-based methods in terms of reduction of exposure to hypoglycemia, especially during the night period (0 am–6 am). In the variance scenario, during the night, an improvement in the time in the target glycemic range (70–180 mg/dL) from 69.0% to 86.4% and a decrease in the time in hypoglycemia (<70 mg/dL) from 10.7% to 2.6% was observed. Robustness analysis showed that the method performance is non-sensitive to carbohydrate records.

**Conclusion:**

The use of CGM and SIP in people with T1D using MDI therapy has the potential to inform smart insulin titration algorithms that improve glycemic control. Clinical studies in real-world settings are warranted to further test the proposed titration algorithm.

**Significance:**

This algorithm is a step towards a decision support system that improves glycemic control and potentially the quality of life, in a population of individuals with T1D who cannot benefit from the artificial pancreas system.

## 1 Introduction

In type 1 diabetes (T1D), life-long insulin replacement is required to compensate for the practically nonexistent insulin secretion due to the autoimmune destruction of the pancreatic beta-cells (1). Without endogenous insulin, glucose regulation is a challenging task, as it is heavily dependent on multiple daily treatment decisions by the patient to account for a wide variety of factors influencing insulin demand, e.g., circadian rhythms, physical activity, food, and stress. Consequently, most patients run the risk of developing long-term micro-/macro-vascular complications due to sustained hyperglycemia (2, 3). Tight glucose control is key to avoiding long-term complications, but fear of hypoglycemia due to overdosing on insulin remains a limiting factor (4). In recent years, technological advances in glucose monitoring devices (5), modern insulin analogs (6), and insulin delivery systems have facilitated T1D management (7, 8). Nevertheless, T1D patients are still not achieving their glycemic targets (9), with complication rates and excess mortality significantly higher in T1D compared to the general population (10).

Worldwide, most T1D patients implement insulin therapy through multiple daily injections (MDI) of insulin (11). Insulin injections comprise two types of insulin formulations: (i) a long-acting analog (e.g., insulin detemir, glargine, or degludec) to maintain glucose levels constant during fasting conditions and overnight, accounting for about 50% of daily insulin requirements; (ii) a rapid-acting analog (e.g., insulin lispro, aspart or glulisine) at mealtimes to compensate for the glycemic excursions due to the macronutrient content of the ingested food or as a correction for hyperglycemia (12). Long-acting insulins, also called basal insulins, are characterized by a slow, often peak-less, absorption with an action duration of up to 24 hours (13). For maximum glucose-lowering effect, basal insulins are typically injected at a consistent time, once or twice a day (e.g., every morning before breakfast and/or every night before bedtime), by means of a vial and a syringe or more convenient insulin pens. The next generation of insulin pens, called smart insulin pens (SIP), can record the history of previous doses, estimate the insulin on board, and connect with a smartphone (14).

The amount of basal insulin needed by a person is specific to each individual as it represents their insulin need in fasting conditions and overnight. Because of changes in people’s lifestyle and metabolism, this dose is periodically titrated to achieve the desired glycemic goals (1). The standard-of-care clinical approach to basal insulin titration involves adjusting the insulin dosage by few units based on self-monitored blood glucose (SMBG) data measured daily in fasting conditions (i.e., pre-breakfast) (15–18). Continuous glucose monitoring (CGM) devices offer the possibility to record glucose levels almost continuously (e.g., 5-minute sampling time), for the whole day. Algorithms using CGM data have been proposed to adapt therapy parameters as part of decision support systems (19–22). If the CGM system is combined with a SIP and a smartphone application, this integrated technology allows to collect a complete data record which enables algorithmic adjustments of the basal insulin dose to accommodate the daily insulin needs of an individual following MDI therapy.

This manuscript proposes a model-based algorithm that utilizes CGM and SIP records to adapt and individualize the long-acting insulin dose for people with T1D under MDI therapy. The algorithm uses an insulin-glucose model to interpret the data, but does not require users to record their meals, which was a limitation of a previously proposed model-based approach (23). Not requiring carbohydrate records renders the method suitable to serve people using MDI therapy, since most do not perform precise carbohydrate counting (24). The proposed method is shown to outperform traditional SMBG-based approaches for the titration of basal insulin (16), in two *in-silico* experiments performed within the University of Virginia/Padova T1D Simulator (25–27). Further, the robustness of the approach to missing carbohydrate records is assessed by comparing the changes to insulin dose suggested by the algorithm with and without carbohydrate information.

## 2 Methods

### 2.1 Novel Algorithm for Titration of Long-Acting Insulin Using CGM and SIP Data

In MDI therapy, people inject long-acting insulin every day approximatively at the same time, to provide a background insulin concentration that keeps their glucose levels in target during fasting conditions and overnight. Here, the objective is to adapt daily long-acting insulin doses in a cyclic manner (e.g., weekly) using individual glucose and insulin data history. It is assumed that a CGM device records glucose levels and a SIP records the time and size of insulin doses (bolus and basal). It is also assumed that data records are preprocessed for non-valid or empty values and sampled with a fixed sampling time dt into arrays with size n. Glucose data is denoted by G, basal insulin data by U_basal_, and bolus insulin data by U_bolus_. At each cycle c, the optimal basal dose $B_{C}^{\text{opt}}$ is determined from the available data 𝒟 = {G, U_basal_, U_bolus_} and a new long-acting basal dose B_c+1_ is generated from the previously used dose B_c_ by following a run-to-run update rule (28):


(1) $${\text{B}}_{\text{c}+1}=B_{C}+\text{Φ}(B_{C}^{\text{opt}}-B_{C}),$$

where Φ is a saturation and dead-zone nonlinearity function that ensures a safe change in the basal dose while being robust to small changes in $B_{C}^{\text{opt}}$ (23). Φ is defined as:


(2) $$\text{Φ}(\text{x})=\{\begin{array}{cc} 0 & \left|\text{x}\right|<{\text{x}}_{\text{min}}\\ \frac{\text{x}}{\left|\text{x}\right|}{\text{x}}_{\text{max}} & \left|\text{x}\right|>{\text{x}}_{\text{max}}\\ \text{x} & \text{otherwise} \end{array}$$

where X_min_ and X_max_ are function parameters chosen to limit and saturate Φ. A table of symbols is provided in **Appendix A.3**.

To determine the optimal basal dose $B_{C}^{\text{opt}}$ , a model of glucose metabolism capable of describing the glycemic response to meals, bolus insulin, and basal insulin is individualized by model identification on the available glucose traces. A residual metabolic signal that allows to explain the experimental data and describes unmodeled phenomena is computed as additional model input by regularized deconvolution (29). The individualized model is then used to predict the effect of basal insulin changes on glucose fluctuations, in the absence of meal and bolus inputs but in the presence of the residual metabolic signal. Thus, an optimization problem can be formulated and solved to find the optimal basal dose that results on the desired glucose profile in fasting conditions.

#### 2.1.1 Individualization of the Metabolic Model

The metabolic model used within the algorithm can be written as the following discrete-time, linear, time-invariant model:


(3) $$\text{X}(\text{k}+1)=A^{d}\text{X}(\text{k})+{\text{B}}_{\text{basal}}^{\text{d}}{\text{U}}_{\text{basal}}(\text{k})+{\text{B}}_{\text{bolus}}^{\text{d}}{\text{U}}_{\text{bolus}}(\text{k})+{\text{B}}_{\text{meal}}^{\text{d}}{\text{U}}_{\text{meal}}(\text{k})+B_{\omega }^{d}\text{ω}(\text{k})\text{Y}(\text{k})=C^{d}\text{X}(\text{k}),$$

where k is the discrete timestamp; X is the metabolic state vector including plasma glucose concentration, insulin concentration in the subcutaneous space and in plasma, insulin action, and carbohydrate absorption from stomach and gut; Y is the model output coinciding with plasma glucose concentration; and A^d^, ${\text{B}}_{\text{basal}}^{\text{d}},{\text{B}}_{\text{bolus}}^{\text{d}},{\text{B}}_{\text{meal}}^{\text{d}},B_{w}^{d}$ , and C^d^ are state-space matrices describing the interaction between glucose and the system inputs.

For our purposes, a linearized subcutaneous oral glucose minimal model (SOGMM) is employed (29), augmented with a basal dose channel (see **Appendix A.1**):


(4) $$\begin{array}{l} {\text{A}}_{\text{c}}=\left[\begin{array}{ccccccccc} -{\text{S}}_{\text{g}} & -{\text{S}}_{\text{i}}{\text{G}}_{\text{b}} & 0 & 0 & 0 & 0 & 0 & \frac{\text{f }{\text{k}}_{\text{q}1}}{{\text{V}}_{\text{g BW}}} & \frac{\text{f }{\text{k}}_{\text{q}2}}{{\text{V}}_{\text{g BW}}}\\ 0 & -{\text{p}}_{2} & 0 & 0 & 0 & 0 & \frac{{\text{p}}_{2}}{{\text{V}}_{\text{i}}\text{ BW}} & 0 & 0\\ 0 & 0 & -{\text{k}}_{\text{sp}} & 0 & 0 & 0 & 0 & 0 & 0\\ 0 & 0 & {\text{k}}_{\text{sp}} & -{\text{k}}_{\text{a}} & 0 & 0 & 0 & 0 & 0\\ 0 & 0 & 0 & 0 & -({\text{k}}_{\text{c}1}+{\text{k}}_{\text{c}12}) & 0 & 0 & 0 & 0\\ 0 & 0 & 0 & 0 & {\text{k}}_{\text{c}12} & -{\text{k}}_{\text{c}2} & 0 & 0 & 0\\ 0 & 0 & 0 & {\text{k}}_{\text{a}} & {\text{k}}_{\text{c}1} & {\text{k}}_{\text{c}2} & -{\text{k}}_{\text{c}\text{l}} & 0 & 0\\ 0 & 0 & 0 & 0 & 0 & 0 & 0 & -({\text{k}}_{\text{q}1}+{\text{k}}_{\text{q}12}) & 0\\ 0 & 0 & 0 & 0 & 0 & 0 & 0 & {\text{k}}_{\text{q}12} & -{\text{k}}_{\text{q}2} \end{array}\right]\\ \;{\text{B}}_{\text{basal}}^{\text{c}}={\begin{array}{ccccccccc} {}[0 & 0 & \text{kF} & (1-\text{k})\text{F} & 0 & 0 & 0 & 0 & 0] \end{array}}^{\text{T}} \end{array}$$

where A^c^ and ${\text{B}}_{\text{basal}}^{\text{c}}$ represent the continuous counterpart of the state-space matrices A^d^ and ${\text{B}}_{\text{basal}}^{\text{d}}$ ; F, k, k_sp_, k_a_ are basal insulin pharmacokinetics parameters described as follows: F (dimensionless) is the basal insulin bioavailability, k (dimensionless) is the precipitate fraction of the administered dose, k_sp_ (min^−1^) is the rate constant of dissolution from precipitate to soluble state, k_a_ (min^−1^) is the rate constant of insulin absorption to plasma; and the remaining parameters are the same as described in Hughes et al. (29).

In equation (3), the initial state X(0) at time of day t_0_ can be determined by assuming that basal insulin doses were given each day at the same time t_B_, considering the other model inputs to be zero. In other words, X(0) is the steady state resulting from a train of Dirac basal inputs given at t_B_ in the absence of any meals and boluses. A closed form for X(0) is derived in **Appendix A.2**. as:


(5) $$\text{X}(0)=\text{exp}(A^{c}(T-{\text{t}}_{\text{B}}))\;{\left(I-\text{exp}(A^{c}T)\right)}^{-1}B_{C}{\text{U}}_{\text{basal}}$$

The SOGMM model takes the meal input stored in U_meal_ in the form of the amount of carbohydrates in the consumed meals. Since the amount of carbohydrates is unknown, this input is artificially reconstructed following insulin dosing rules (30):


(6) $${\text{U}}_{\text{meal}}(\text{k})=\text{max}\left(\frac{500}{TDI}\left({\text{U}}_{\text{bolus}}(\text{k})-\frac{\text{G}(\text{k})-G_{b}}{1800}TDI\right),0\right)\text{k}\in [1,\text{n}],$$

where TDI is the total daily dose of insulin and G_b_ is basal glucose. According to (6), the input U_meal_ can be nonzero only if the input U_bolus_ is nonzero, thus including only instances of bolused carbohydrate intakes. To include in the input vector unbolused meal events, U_meal_ is further augmented by including meal inputs identified through an unbolused-meal detection algorithm (31, 32).

Model (3) is individualized on patient data 𝒟 = {G, U_basal_, U_bolus_} by estimating one insulin sensitivity parameter (S_i_) per day. Additionally, carbohydrate absorption rate parameters (f,k_q1_,k_q2_,k_q12_) are estimated for each meal to account for inter-meal absorption differences (e.g., from the type and ratio of macronutrients in the consumed meal) and errors in the reconstructed meal input. Population parameters are used for the remaining parameters.

The list of parameters is denoted by $\text{θ}={\left(f^{m},{\text{k}}_{\text{q}1}^{\text{m}},{\text{k}}_{\text{q}2}^{\text{m}},{\text{k}}_{\text{q}12}^{\text{m}},S_{i}^{d}\right)}_{m\in meals\&d\in \text{days}}$ . Parameters are estimated following a maximum-a-posteriori approach where the posterior probability of observing θ conditioned on the data 𝒟 is maximized. Note that in this step the residual metabolic signal (ω) is not considered. The parameter vector is thus obtained as:


(7) $$\hat{\text{θ}}=\text{arg}\underset{\text{θ}}{\text{max}}\text{P}(\text{θ}|\text{G},{\text{U}}_{\text{basal}},{\text{U}}_{\text{bolus}},\text{ω}=0)=\text{arg}\underset{\text{θ}}{\text{max}}\text{P}(\text{G}|\text{θ},{\text{U}}_{\text{basal}},{\text{U}}_{\text{bolus}},\text{ω}=0)\text{P}(\text{θ}),$$

where 𝒟(θ) is derived from a personalized normal prior distribution and 𝒟(G|θ, U_basal_, U_bolus_, ω = 0) is the likelihood of the measurements G calculated by assuming they are independently and identically distributed under a normal distribution with a constant coefficient of variation and mean Y(θ, U_basal_, U_bolus_, ω = 0).

#### 2.1.2 Estimation of the Residual Metabolic Signal

The residual metabolic signal ω is estimated by regularized deconvolution *via* inversion of the individualized model outlined above. The deconvolution procedure is described by Patek et al. (33).

#### 2.1.3 Optimization of Long-Acting Insulin

The individualization procedure and the residual metabolic signal estimation are performed daily using an extended daily data (a day padded by a 6-hour head and a 2- hour tail before and after the day). Using the identified model, the glucose trace in response to the basal dose, in the absence of meals and insulin boluses, can be predicted as:


(8) $${\text{X}}_{\text{basal}}(\text{k}+1)=A^{d}{\text{X}}_{\text{basal}}(\text{k})+{\text{B}}_{\text{basal}}^{\text{d}}{\text{U}}_{\text{basal}}(\text{k})+B_{\omega }^{d}\text{ω}(\text{k}){\text{Y}}_{\text{basal}}(\text{k})=C^{d}{\text{X}}_{\text{basal}}(\text{k}),$$

The basal dose can then be optimized to achieve a desired theoretical fasting glucose profile Y_basal_. For this purpose, the glycemic risk during the day (midnight to midnight) is minimized using a risk function assuming higher values when glucose is <70 mg/dL and >180 mg/dL (34). Protection against hypoglycemia is reinforced by further penalizing glucose levels under the desired target G_t_ (set to 110 mg/dL) during the night period ℐ_night_, personalized to the patient data 𝒟 by searching for periods of time without insulin boluses. The optimization problem can be written as follows:


(9) $$B_{C}^{\text{opt}}=\text{arg}\underset{{\text{U}}_{\text{basal}}}{\text{min}}\left\{\sum_{\text{k}}risk({\text{Y}}_{\text{basal}}(\text{k}))+\text{α}\sum_{\text{k}\in {\text{J}}_{\text{night}}}\text{max}\left(1-\frac{{\text{Y}}_{\text{basal}}(\text{k})}{{\text{G}}_{\text{t}}},0\right)\right\},$$

and is solved *via* grid search performed around the currently used basal dose B_c_, testing changes up to ±40%, with a fixed 1% step size. If multiple days are analyzed simultaneously, $B_{C}^{\text{opt}}$ is found for each day and then averaged over days. The final optimal dose is rounded to the nearest half unit, to accommodate SIP resolution. This algorithm can be run routinely at night whenever the daily data is collected.

### 2.2 State-of-the-Art Algorithms for Basal Insulin Titration Using SMBG Data

Currently, most patients using MDI therapy do not own a SIP nor consistently use a CGM, even though these technologies are getting cheaper and more accessible (5) (35). Long-acting insulin can still be titrated and adapted using SMBG values from a glucose meter. SMBG is taken in fasting conditions, usually before the breakfast meal, and used to adjust the basal insulin dose. In the following, two existing strategies to adapt the basal insulin dose using SMBG records will be presented and compared against the proposed algorithm.

For each cycle c, collected SMBG values, denoted ${\left({\text{G}}_{\text{basal}}^{\text{i}}\right)}_{\text{i}\in [1,\text{C}]}$ , will be used to compute the new optimal basal dose $B_{C}^{\text{opt}}$ . In order to keep a fair comparison between the algorithms, the same update rule, with the same saturation and dead-zone, reported in equation (1) is used but with the newly calculated $B_{C}^{\text{opt}}$ .

#### 2.2.1 Control to Range

Long-acting insulin dose can be titrated following a control-to-range heuristic rule. Rules are composed of a chosen range (e.g., 80-130 mg/dL) and an insulin adjustment step (e.g., 1U). Most titration algorithms used in clinical practice can be considered of this sort (36). Here, an algorithm similar to the one proposed by Visentin et al. to titrate insulin glargine was used (37), which defines the optimal insulin dose as:


(10) $${\text{B}}_{\text{c}}^{\text{opt}}=\{\begin{array}{l} \begin{array}{cc} {\text{B}}_{\text{c}}-\Delta {\text{b}}_{\text{d}} & \underset{i\in [1,\text{C}]}{\text{min}}\left({\text{G}}_{\text{basal}}^{\text{i}}\right)<{\text{G}}_{\text{min}} \end{array}\\ \begin{array}{cc} {\text{B}}_{\text{c}}-\Delta {\text{b}}_{\text{i}} & \frac{1}{\text{C}}\underset{i\in [1,\text{C}]}{\Sigma }\left({\text{G}}_{\text{basal}}^{\text{i}}\right)<{\text{G}}_{\text{max}} \end{array}\\ \begin{array}{rr} {\text{B}}_{\text{c}} & \text{Otherwise} \end{array} \end{array}$$

where G_min_ = 80; G_max_ = 130; Δb_d_ is 10% of B_c_ and is at least 1U; and Δb_i_ is ranging from 0.5U to 5U, with 0.5U step every 20 mg/dL.

#### 2.2.2 Control to Reference

This baseline algorithm is taken from recent work from Cescon et al. where an iterative learning control (ILC) is proposed to optimize the basal insulin dose (16). This ILC algorithm finds the new optimal dose $B_{C}^{\text{opt}}$ to be administered such that the SMBG values are driven as close as possible to the desired reference trajectory G_target_:


(11) $$B_{C}^{\text{opt}}=B_{C}+\gamma F(\text{q})\;({\text{G}}_{\text{target}}-\frac{1}{\text{C}}\sum_{\text{i}\in [1,\text{C}]}{\text{G}}_{\text{basal}}^{\text{i}}),$$

where γ is a gain set to 1, G_target_ is set to 110 mg/dL, and F(q) is a discrete filter described in (16). Similarly to (16), the DC gain of the filter F(q) is individualized to each subject as $\frac{3}{\text{BW}}$ .

### 2.3 *In-Silico* Studies and Outcome Metrics

The basal insulin dosing adaptation algorithm was tested in a 120-day simulation study including 100 virtual adult subjects with T1D using MDI therapy. The simulation experiment was carried on in the FDA-accepted University of Virginia/Padova T1D Simulator. Daily basal insulin doses of glargine-100 were administered either in the morning or before bedtime and were usually taken around mealtime, when possible (i.e., if a meal is programmed at the same time as the programmed time interval when the basal dose is taken, the basal dose is given at the same time as the meal). Bolus doses accompanied announced meals and were calculated using the counted carbohydrates, glucose at mealtime, glucose target (set to 110 mg/dL), and the subject carbohydrate ratio and insulin sensitivity factor. The virtual subjects could treat hypoglycemia events, but a hypoglycemia unawareness algorithm was implemented where there is a chance that the hypoglycemia event is not treated for a period (e.g., for each minute while glucose is under 70 mg/dL there is a 10% chance that the hypoglycemia is treated, equivalent to 4% chance hypoglycemia lasts for >30min). Additionally, hypoglycemia events between 0 am at 6 am were not treated to reinforce night hypoglycemia.

Each virtual subject was characterized by a basal glucose and the basal dose needed to achieve this basal glucose, ${\text{U}}_{\text{basal}}^{\text{ss}}$ . The starting basal dose was altered from ${\text{U}}_{\text{basal}}^{\text{ss}}$ by +50% for half of the subjects and -50% for the other half. The carbohydrate ratio was not optimized but slightly altered to keep a reasonable total daily insulin dose: the bolus dose was decreased by 25% for subjects w here the basal dose was increased by 50% and vice-versa. As reported in (27), a model for glucose meter was used to generate SMBG values from blood glucose levels, and a model for a CGM sensor was used to generate glucose readings (38). Consumed carbohydrate amount was predetermined in the simulator but unknown to the adaptation algorithms.

Five treatment arms were simulated: a) a control arm where altered insulin dosing parameters were kept the same throughout the experiment (CTR); b) a baseline arm where the control to range SMBG algorithm is employed (SMBG-Rule); c) a baseline arm where the control to reference SMBG algorithm is employed (SMBG-ILC); d) an experimental arm where the proposed algorithm is employed (CGM-Opt); e) another experimental arm similar to d) but where counted carbohydrate amount was given to the optimization algorithm instead of being reconstructed (CGM-Opt-Carb). Treatment arms for each virtual subject were identical, with the same meals and metabolic/behavioral variability.

Similar to clinical practice (39), the insulin dose was titrated every three days to provide enough time for insulin dose change effects on fasting glucose to stabilize. In the SMBG-Rule and SMBG-ILC arms, three pre-breakfast SMBG values were used. In the CGM-opt and CGM-Opt-Carb arm, the CGM data up to the time of the next basal dose recommendations (around three days) was used. The simulation experiment was not interrupted during the 120 days. The same simulation was repeated for two different scenarios: *nominal* and *variance*, described below.

#### 2.3.1 *Nominal* Scenario

In this scenario, the virtual subjects consumed three similar meals each day at the same time. Meals were taken at 7AM, 1PM, 7PM and the amount of carbohydrates per meal was 50g, 75g and 75g. There was no additional metabolic/behavioral variability.

#### 2.3.2 *Variance* Scenario

In this scenario, metabolic/behavioral variability was implemented. Behavioral variability consisted of consuming three main meals and up to three unannounced and unbolused snacks. Meal sizes were variable, but the total carbohydrates consumed over the day were between 200g and 300g, main meals were bigger than 30g, and snacks were smaller than 40g (e.g., three main meals 50g, 70g, 60g and two snacks 20g, 20g). The carbohydrate amount used in the insulin bolus calculation was subject to a random carbohydrate counting error uniformly distributed between -40% and +40%. The insulin bolus could be delayed by up to 1 hour after consuming a meal. Metabolic variability was implemented by varying the insulin sensitivity during the day and between days.

#### 2.3.3 Outcome Metrics

Algorithm performance was assessed using established metrics of glycemic variability and quality of glycemic control (40), including, time in the target range of 70-180 mg/dL (TIR); time in hypoglycemia <70 mg/dL (TBR); and time in hyperglycemia >180 mg/dL (TAR) (A complete list of outcome metrics are listed in **Table 1**). Metrics were calculated for each arm every 15 days. Before starting the 120 days experiment, an initial 15 days was simulated and used as a baseline. All results are reported as mean ± standard deviation across subjects for a 15-day duration. Changes of a certain 15-day period as compared to the baseline period are reported as average and confidence interval (CI).

**Table 1:** Summary of glycemic outcomes for the in-silico experiment.

			Baseline 15 days before start	CTR last 15	SMBG-Rule last 15 days	SMBG-ILC last 15 days	CGM-Opt last 15 days
***Nominal***	**Overall**	**Time in 70-180 (mg/dL)**	68.2 (18.3)	**67.4 (18.5)**	71.2 (17.7)	**74.2 (17.2)**	68.4 (17.5)
		**Time in 70-140 (mg/dL)**	41.4 (19.3)	40.7 (19.3)	45.4 (16.5)	**49.3 (15.1)**	**40.2 (12.0)**
		**Time < 70 (mg/dL)**	5.6 (8.4)	5.6 (8.5)	2.1 (4.4)	2.2 (4.1)	**0.5 (1.5)**
		**Time < 54 (mg/dL)**	2.9 (5.7)	3.0 (5.8)	0.5 (1.5)	0.5 (1.3)	**0.1 (0.6)**
		**Time > 180 (mg/dL)**	26.2 (18.3)	27.0 (18.7)	26.6 (18.5)	**23.6 (17.9)**	**31.0 (17.9)**
		**Time > 250 (mg/dL)**	3.2 (5.7)	3.4 (5.8)	3.7 (6.9)	**3.3 (6.7)**	**5.0 (8.4)**
		**Mean (mg/dL)**	147.7 (25.4)	148.5 (25.9)	150.5 (23.8)	145.4 (23.1)	160.5 (20.6)
		**Standard deviation (mg/dL)**	43.0 (14.0)	**43.4 (14.0)**	41.7 (12.5)	41.7 (12.6)	**40.4 (11.9)**
		**Total insulin (U)**	49.1 (24.7)	49.2 (24.7)	48.4 (23.2)	51.4 (25.2)	43.5 (20.1)
		**Basal insulin (U)**	28.6 (22.6)	28.6 (22.6)	27.8 (19.4)	31.3 (20.2)	21.7 (14.2)
		**Bolus insulin (U)**	20.5 (11.0)	20.5 (11.1)	20.6 (10.6)	20.1 (10.0)	21.8 (10.7)
	**Night [0am–6am]**	**Time in 70-180 (mg/dL)**	82.8 (25.3)	**82.6 (25.5)**	92.8 (15.0)	93.9 (12.6)	**98.6 (3.8)**
		**Time in 70-140 (mg/dL)**	64.9 (31.5)	**65.0 (31.3)**	81.6 (19.9)	**87.3 (15.4)**	86.6 (13.2)
		**Time < 70 (mg/dL)**	15.2 (25.8)	**15.4 (26.1)**	5.8 (15.1)	5.1 (12.4)	**0.2 (1.0)**
		**Time < 54 (mg/dL)**	8.4 (18.1)	**8.6 (18.4)**	1.3 (5.6)	1.0 (3.4)	**0.0 (0.1)**
		**Time > 180 (mg/dL)**	2.0 (5.0)	**2.0 (5.0)**	1.4 (3.7)	**1.0 (3.7)**	1.2 (3.7)
		**Time > 250 (mg/dL)**	0.0 (0.0)	**0.0 (0.0)**	**0.0 (0.0)**	**0.0 (0.0)**	**0.0 (0.0)**
		**Mean (mg/dL)**	108.3 (30.7)	108.0 (30.6)	110.5 (21.8)	104.6 (17.4)	122.6 (8.3)
		**Standard deviation (mg/dL)**	16.9 (8.2)	**17.1 (8.2)**	15.3 (6.3)	15.0 (6.2)	**14.9 (6.1)**
	**Pre-breakfast SMBG(mg/dL)**		113.6 (38.9)	113.2 (39.1)	117.5 (19.0)	111.6 (6.2)	130.2 (14.1)
***Variance***	**Overall**	**Time in 70-180 (mg/dL)**	57.4 (14.6)	**57.4 (14.4)**	63.9 (14.4)	**65.0 (12.4)**	63.6 (15.4)
		**Time in 70-140 (mg/dL)**	30.2 (13.8)	**30.1 (13.6)**	38.9 (13.3)x	**44.5 (13.3)**	36.7 (14.2)
		**Time < 70 (mg/dL)**	4.5 (5.2)	4.6 (5.5)	5.3 (5.4)	**9.8 (7.1)**	**2.8 (2.9)**
		**Time < 54 (mg/dL)**	2.5 (3.5)	2.4 (3.7)	2.6 (3.1)	**5.0 (4.1)**	**1.3 (1.5)**
		**Time > 180 (mg/dL)**	38.1 (15.7)	38.0 (15.6)	30.9 (15.6)	25.2 (14.3)	33.5 (16.9)
		**Time > 250 (mg/dL)**	11.5 (8.7)	11.1 (8.5)	9.1 (7.8)	**7.3 (6.8)**	10.5 (8.8)
		**Mean (mg/dL)**	169.8 (25.3)	169.1 (25.3)	158.8 (24.8)	144.9 (25.0)	165.8 (26.0)
		**Standard deviation (mg/dL)**	60.7 (14.6)	**59.8 (14.1)**	58.1 (14.3)	58.8 (14.8)	**56.8 (13.5)**
		**Total insulin (U)**	51.8 (22.6)	52.0 (22.7)	57.9 (25.7)	67.1 (32.8)	55.1 (26.5)
		**Basal insulin (U)**	28.2 (18.5)	28.2 (18.5)	35.0 (18.4)	45.4 (25.8)	31.7 (17.9)
		**Bolus insulin (U)**	23.5 (13.2)	23.8 (13.1)	22.9 (11.7)	21.8 (10.8)	23.5 (11.4)
	**Night [0am–6am]**	**Time in 70-180 (mg/dL)**	68.5 (18.9)	**69.0 (19.8)**	80.2 (14.5)	72.9 (14.2)	**86.4 (9.9)**
		**Time in 70-140 (mg/dL)**	42.8 (24.7)	**41.9 (24.2)**	59.4 (18.4)	**61.1 (14.9)**	59.4 (14.7)
		**Time < 70 (mg/dL)**	10.9 (16.9)	10.7 (17.3)	9.8 (13.3)	**20.6 (16.3)**	**2.6 (3.3)**
		**Time < 54 (mg/dL)**	7.0 (12.3)	6.5 (12.4)	5.2 (8.4)	**10.9 (10.7)**	**1.1 (2.0)**
		**Time > 180 (mg/dL)**	20.6 (20.6)	**20.4 (21.1)**	10.1 (12.6)	**6.5 (9.0)**	11.0 (9.6)
		**Time > 250 (mg/dL)**	3.1 (5.3)	**2.5 (4.9)**	1.6 (3.5)	**1.0 (2.6)**	1.7 (3.2)
		**Mean (mg/dL)**	138.5 (38.1)	138.3 (38.1)	125.1 (26.6)	107.0 (24.9)	135.9 (13.7)
		**Standard deviation (mg/dL)**	39.5 (14.3)	**37.1 (11.7)**	36.0 (11.8)	36.4 (11.7)	**35.7 (12.0)**
	**Pre-breakfast SMBG(mg/dL)**		144.4 (37.1)	144.5 (37.8)	132.3 (10.6)	115.0 (7.5)	138.0 (18.1)

Values are reported as mean and standard deviation. Best outcomes are in green and worst in red.

## 3 Results

**Figure 1:**
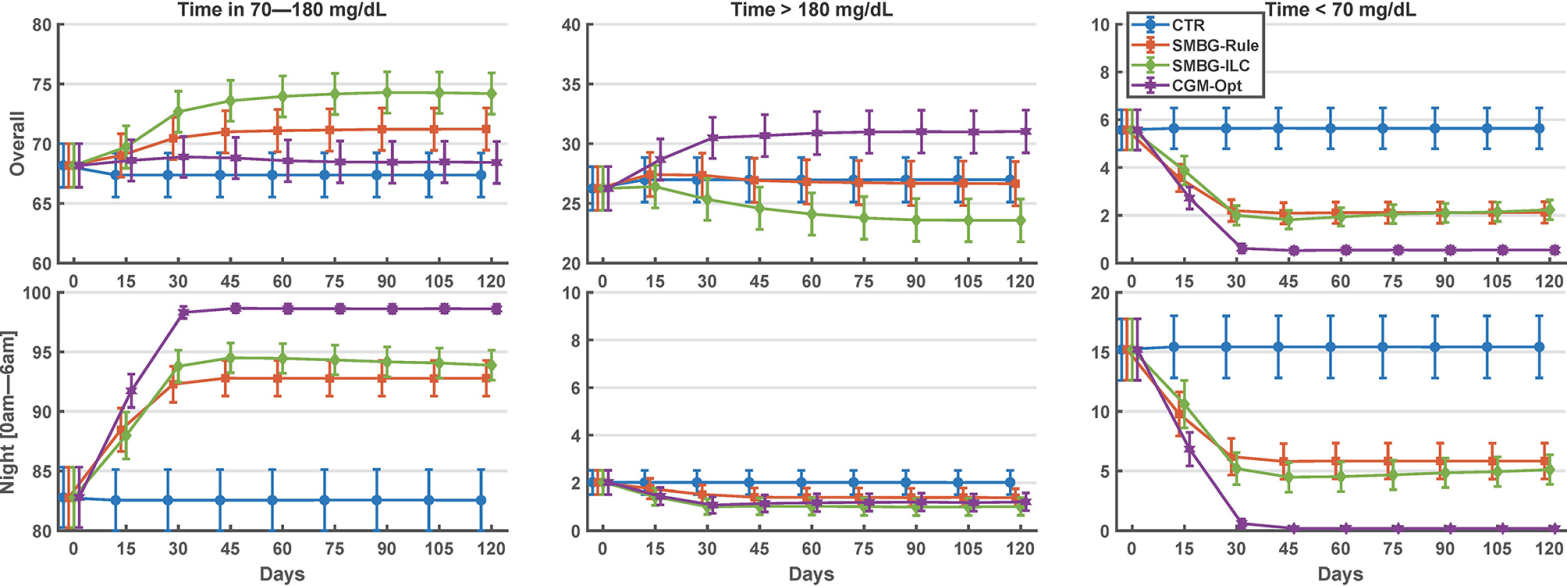
Summary of glycemic outcomes in every 15 days period for the nominal scenario of the in-silico experiment. Values are shown as mean and standard deviation.

**Figure 2:**
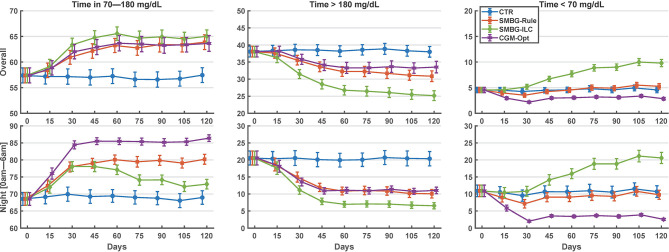
Summary of glycemic outcomes in every 15 days period for the variance scenario of the in-silico experiment. Values are shown as mean and standard deviation.

Comparing the last 15 days of the 120-day simulation to the baseline period, the CGM-Opt did not change TIR in the *nominal* scenario (+1.0% CI(-1.5% to 3.6%)), but increased TIR from 57.4% (±14.6%) to 63.6% (±15.4%) by +6.2% CI(3.6% to 8.8%) in the *variance* scenario. At the same time, TBR was decreased using the CGM-Opt in both scenarios (-5.1% CI(-6.9% to -3.4%) in the *nominal* scenario, and -1.7% CI(-3.0% to -0.4%) in the *variance* scenario). The CGM-Opt performed exceptionally well during the night period, where TIR increased by +16.1% CI(11.0% to 21.1%) in the *nominal* scenario and by +17.4% CI(13.8% to 21.0%) in the *variance* scenario, while TBR was decreased by -14.0% CI(-19.0% to -8.9%) in the *nominal* scenario and by -8.1% CI(-11.6% to -4.6%) in the *variance* scenario. These results are summarized in **Figure 1** for the *nominal* scenario and **Figure 2** for the *variance* scenario. An exhaustive comparison between the four arms (CTR, SMBG-Rule, SMBG-ILC, and CGM-Opt) is provided in **Table 1**.

**Figure 3:**
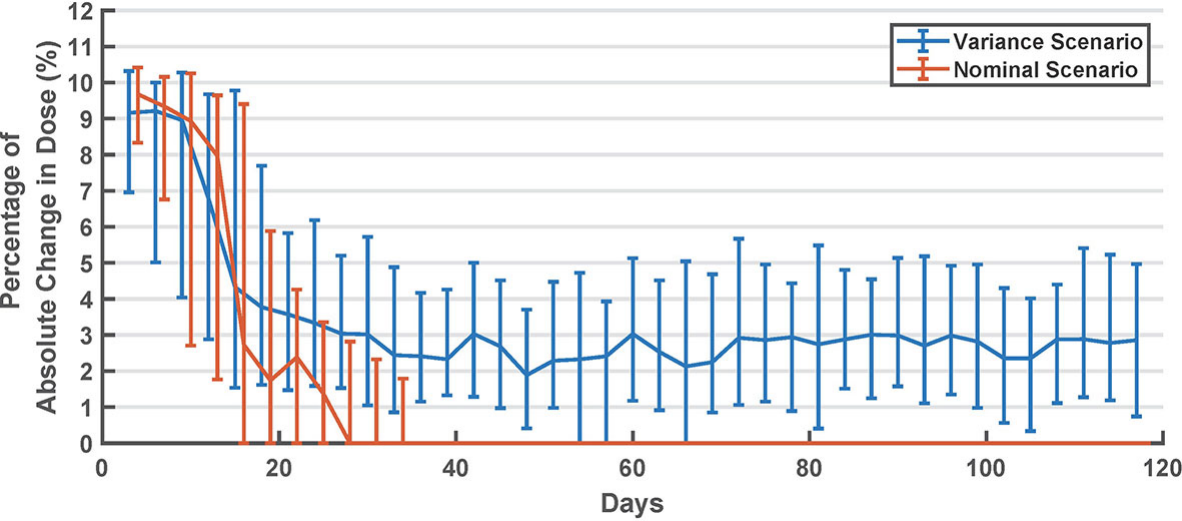
Summary of long-acting dose changes in titration days (every 3 days). Values are shown as median and interquartile range.

The average basal dose in the last 15-days was compared to the original ${\text{U}}_{\text{basal}}^{\text{ss}}$ . In **Table 2**, results of this comparison are shown separately for virtual subjects that started with a higher basal dose and with a lower basal dose. In **Figure 3**, the percentage basal dose changes in both scenarios are shown.

**Table 2:** Summary of changes in basal dose from theoretical steady-state optimal value.

	*Change in basal insulin from theoretical optimal*	*CTR*	*SMBG-Rule*	*SMBG-ILC*	*CGM-Opt*
*Nominal*	Baseline with higher dose (n=50)	14.2U (8.1) 50.4% (0.6)	8.7U (8.2) 31.7% (20.9)	6.5U (8.2) 25.0% (25.4)	-4.2U (8.1) -11.3% (24.8)
	Baseline with lower dose (n=50)	-14.7U (7.7) -49.4% (0.7)	-10.7U (6.3) -37.7% (13.8)	-1.6U (10.0) -7.9% (29.3)	-10.2U (10.4) -33.8% (26.7)
*Variance*	Baseline with higher dose (n=50)	13.8U (5.3) 50.5% (0.6)	11.2U (9.8) 40.4% (30.5)	17.9U (13.4) 65.3% (41.7)	3.9U (7.2) 14.1% (29.9)
	Baseline with lower dose (n=50)	-15.1U (9.7) -49.5% (0.7)	1.5U (8.4) 11.9% (31.9)	15.8U (15.8) 57.1% (40.5)	2.5U (10.2) 13.0% (30.2)

Results are reported as mean and standard deviation of absolute differences and percentage differences, respectively.

There was no clinically significant change in the glycemic metrics between the CGM-Opt arm and the CGM-Opt-Carb arm. Between the two arms the difference in glycemic outcomes in the last 15 days were: TIR differed by +0.1% CI(-0.1% to 0.3%) and TBR by 0.0% CI(-0.0% to 0.01%) in the *nominal* scenario; and TIR differed by +0.2% CI(0.0% to 0.4%) and TBR by 0.0% CI(-0.1% to 0.1%) in the *variance* scenario. Similarly, as it can be seen in **Table 3**, there was no differences between calculated optimal basal dose $B_{C}^{\text{opt}}$ for each day in the two arms (CGM-Opt vs CGM-Opt-Carb).

**Table 3:** Summary of changes between the optimization procedure when the carbohydrate input is reconstructed or counted by virtual subjects.

	*Absolute differences averaged per subject (100 subjects)*	*Absolute differences averaged per change (39 changes)*
*Nominal scenario*	0.2U (0.4) [0 to 1.8]	0.2U (0.04) [0.2 to 0.3]
*Variance scenario*	0.4U (0.5) [0 to 2.7]	0.4U (0.1) [0.2 to 0.7]

Values are reported as mean, standard deviation, minimum, and maximum.

## 4 Discussion

People with T1D live with the life-long burden of making important decisions about their daily insulin doses. Technological advances in diabetes treatment can help in easing this burden. Specifically, the new generation of SIP and the affordability of CGM are facilitating the development of a decision support system designed for people using MDI therapy. The proposed algorithm will enable such decision support systems by automatically suggesting adaptation of the basal insulin dose after analyzing SIP and CGM records.

Our algorithm is based on a metabolic model that describes the complex glucose traces by separating the effects of the basal insulin dose from other system inputs, i.e., insulin boluses and consumed meals. This approach is inspired by the clinical practice where patients are usually asked to skip meals in order to optimize their basal insulin (41). Once the model is able to describe the data, we can mathematically eliminate the effect of meals and boluses on the glucose trace, thus isolating the effect of basal dose on the theoretical fasting glucose and allowing for its optimal tuning. This method follows a similar insulin basal rate optimization approach described by Fabris et al. (42). Estimating the residual metabolic signal is key to our approach since it detects changes in the glucose curve that are independent of delivered insulin boluses and consumed meals but needs to be controlled through the basal dose. The original idea of a model-based residual metabolic signal estimated for insulin titration was introduced by Patek et al. and refined in other works (29, 33, 43). Another similar model-based method was proposed previously by El Fathi et al., but it employed a simpler model to describe the basal dose absorption and action on glucose (23). In this work, a recent subcutaneous absorption model of basal dose was employed (44). Another difference is that the basal dose is optimized independently from other model parameters, giving the possibility to mold the cost function to enforce a desired outcome (e.g., increased hypoglycemia protection at night.).

To put the performance of this algorithm into perspective, we compared it with a control-to-range algorithm inspired by the current clinical practice and a control-to-reference algorithm that was recently proposed. Both algorithms use the current standard of titrating the long-acting insulin dose from the pre-breakfast SMBG measurement. In general, results have shown that the proposed algorithm outperforms the other methods at night and can achieve comparable results overall. This can be explained by multiple factors (i) with CGM, we can observe the full glucose profile, thus clearly detecting degradations in night control; (ii) we explicitly biased the optimization equation in (9) to reduce hypoglycemia events during the night; (iii) once the night period is optimized, we did not aim to optimize glycemic metrics in the day period by optimizing insulin boluses. Our algorithm also reduced glycemic variability as measured by the glucose standard deviation in both scenarios (**Table 1**). This is aligned with our observation in **Table 2** that this algorithm can recover the theoretical steady-state basal dose, which in theory is the one that will cause the least variations in the glucose curve. Furthermore, one can argue that reducing overall glycemic variability will facilitate optimizing parameters used to compute insulin boluses in the day period.

Our simulations have shown that the control-to-range algorithm used in the clinical practice (SMBG-Rule) is effective in titrating the long-acting insulin dose by reducing both hypoglycemia and hyperglycemia. Unexpectedly, the control-to-reference algorithm (SMBG-ILC) did not perform similarly in the two scenarios. In spite of showing good performance in the *nominal* scenario, the ILC-based algorithm was not able to reduce hypoglycemia in the *variance* scenario. In **Table 1**, we can see that the mean fasting SMBG values were driven close to 110 as expected by the algorithm, but this was achieved at the cost of a higher hypoglycemia exposure. This suggests that individualizing the target of the ILC algorithm (or stopping titration early) for each subject may be necessary in clinical practice. This simulation also hints that the ILC algorithm may benefit from the use of CGM data instead of SMBG.

We have also shown that our algorithm is robust to carbohydrate information, as seen in **Table 3**. This is a result of keeping meal parameters free to describe the glucose curve with the least *a-priori* knowledge during the model individualization. Therefore, the proposed meal reconstruction approach using the simple equation in (6) is shown to be sufficient for titration purposes. Not relying on carbohydrate counting will facilitate the use of this algorithm by T1D patients.

In **Figure 3**, we can see that the algorithm converges in about 30 days (after ~10 cycles). However, in the *variance* scenario, the algorithm continued to make small changes. This can be attributed to the metabolic and behavioral variability in this scenario. In this scenario, the algorithm might be undesirably chasing noise, which suggests that the dead zone in Eq. 2 can be tuned to reduce this effect. Interestingly, as seen in **Figure 2**, glycemic outcomes are kept stable even after these changes.

We should recognize that our results are limited by the type of scenarios we chose and the capabilities of the simulation platform, mainly the amount and frequency of the metabolic and behavioral variabilities. These results are also limited by the chosen cost function in Eq. 9 which is biased towards decreasing the hypoglycemia exposure risk during the night period, and neglecting the daytime period. This algorithm may be combined with an insulin bolus optimization algorithm that optimize glycemic outcomes during the daytime period.

## 5 Conclusions

This paper introduces a novel algorithm to titrate long-acting insulin doses in individuals with T1D following MDI therapy and using CGM and SIP. With the quick rise of CGM use and the arrival of SIP, the need for such algorithms is warranted. Our proposed method did not require carbohydrate information, and a proof-of-concept *in*-*silico* study demonstrated that the method performs well in simulation, increasing time spent in the target range, while reducing exposure to hypoglycemia, hyperglycemia, and glycemic variability. This algorithm will be evaluated as part of a decision support system in an upcoming clinical trial with people with T1D using MDI therapy (NCT04443153).

## Data Availability Statement

The raw data supporting the conclusions of this article will be made available by the corresponding author upon reasonable request, without undue reservation.

## Author Contributions

AE developed the algorithm, performed the *in-silico* experiments and drafted the manuscript. CF and MBD contributed to the theoretical development of the algorithm and reviewed the manuscript. All authors contributed to the article and approved the submitted version.

## Funding

This work is supported by NIH-NIDDK project R01DK011562.

## Conflict of Interest

MBD receives research support from Tandem Diabetes, Dexcom, Novo Nordisk, and Arecor paid to his institution. MDB serves as a consultant for Tandem, Dexcom, Adocia, Air Liquide, and Roche. MBD received speaker fees from Tandem and Arecor.

The remaining authors declare that the research was conducted in the absence of any commercial or financial relationships that could be construed as a potential conflict of interest.

## Publisher’s Note

All claims expressed in this article are solely those of the authors and do not necessarily represent those of their affiliated organizations, or those of the publisher, the editors and the reviewers. Any product that may be evaluated in this article, or claim that may be made by its manufacturer, is not guaranteed or endorsed by the publisher.

## Appendix

### A.1 Long-acting Insulin Model Integration in the UVA/Padova T1D Simulator

A long-acting insulin model describing subcutaneous absorption was implemented following work by Schiavon et al. (44). This is a two compartment model described by the following differential equations:

$$\begin{array}{l} \begin{array}{cc} \frac{\text{d}}{\text{dt}}{\text{I}}_{\text{q}1}(\text{t})=-{\text{k}}_{\text{sp}}{\text{I}}_{\text{q}1}(\text{t})+\text{k}\;\text{F}\;{\text{U}}_{\text{basal}} & {\text{I}}_{\text{q}1}(0)=0 \end{array}\\ \begin{array}{cc} \frac{\text{d}}{\text{dt}}{\text{I}}_{\text{q}2}(\text{t})=-{\text{k}}_{\text{a}}{\text{I}}_{\text{q}2}(\text{t})+{\text{k}}_{\text{sp}}{\text{I}}_{\text{q}1}(\text{t})+(1-\text{k})\text{F}\;{\text{U}}_{\text{basal}} & {\text{I}}_{\text{q}2}(0)=0 \end{array}\\ \text{R}{\text{a}}_{\text{I}}(\text{t})={\text{k}}_{\text{a}}{\text{I}}_{\text{q}2}(\text{t}) \end{array}$$

where U_basal_ (mU/kg/min) is the glargine insulin dose administered into the subcutis, F (dimensionless) the glargine bioavailability, k (dimensionless) the precipitate fraction of the administered dose, k_sp_ (min^−1^) the rate constant of dissolution from precipitate to soluble state, k_a_ (min^−1^) the rate constant of insulin absorption to plasma, and R_aI_ is the insulin rate of appearance into plasma.

Model parameters of the long-acting insulin Glargine-100 were used in the simulator. Parameters values were randomly drawn from the reported empirical distribution by Visentin et al. (37). Correlation between parameters were not considered.

### A.2 Steady-State Solution for MDI Virtual Subject

In here, we assume that the basal dose is given at the same time t_B_ each day (day duration is T). We assume a line time invariant state space model where the basal dose is considered as a Dirac input, and we ignore all other inputs to the system. At day 1, the state at time t can be written as:

$$\text{X}(\text{t})=\text{exp}(A^{c}\text{t})\text{X}(0)+\int_{0}^{\text{t}}\text{exp}(A^{c}(\text{t}-\text{τ}))B_{C}{\text{U}}_{\text{basal}}(\text{t})\text{d}\tau$$

Since U_basal_ (t) = U_basal_ δ(t = t_B_), we have for t ≥t_B_

$$\text{X}(\text{t})=\text{exp}(A^{c}\text{t})\text{X}(0)+\text{exp}(A^{c}(\text{t}-{\text{t}}_{\text{B}}))B_{C}{\text{U}}_{\text{basal}}$$

At day n, for ${\text{t}}_{\text{B}}+\left(n-1\right)T\leq t<nT$ , we assume that basal injections have happened at {tB + kT}k_∈[0,n – 1]_, and the current time is t_0_ + nT.

$$\text{X}(\text{t})=\text{exp}({\text{A}}^{\text{c}}\text{t})\text{X}(0)+\sum_{\text{k}=0}^{\text{n}-1}\text{exp}\left({\text{A}}^{\text{c}}\left({\text{t}}_{0}+n\text{T}-({\text{t}}_{\text{B}}-\text{k}\text{T})\right)\right){\text{B}}_{\text{c}}{\text{U}}_{\text{basal}}$$

Which can be written as,

$$\text{X}(\text{t})=\text{exp}({\text{A}}^{\text{c}}\text{t})\text{X}(0)+\text{exp}({\text{A}}^{\text{c}}({\text{t}}_{0}-{\text{t}}_{\text{B}}))\left(\sum_{\text{k}=0}^{\text{n}-1}\text{exp}\left((\text{n}-\text{k}){\text{A}}^{\text{c}}\text{T}\right)\right){\text{B}}_{\text{c}}{\text{U}}_{\text{basal}}$$

By performing the variable change k' = n – k.

X(t)=exp(Act)X(0)+exp(Ac(t0−tB))exp(AcT)(∑k'=0n−1exp(k'AcT)BcUbasal

Or, since A^c^ is marginally stable,

$$\text{X}(\text{t})=\text{exp}({\text{A}}^{\text{c}}\text{t})\text{X}(0)+\text{exp}({\text{A}}^{\text{c}}({\text{t}}_{0}-{\text{t}}_{\text{B}}+\text{T}))\;(\text{I}-\text{exp}(\text{n}{\text{A}}^{\text{c}}\text{T})){\;(\text{I}-\text{exp}({\text{A}}^{\text{c}}\text{T}))}^{-1}{\text{B}}_{\text{c}}{\text{U}}_{\text{basal}}$$

When t = t_0_ +nT is big enough, we have exp(A^c^ t) ~0 and exp(nA^c^ T) ~0. Thus, the above expression simplifies to:

$$\text{X}({\text{t}}_{0})=\text{exp}\;({\text{A}}^{\text{c}}(\text{T}-({\text{t}}_{\text{B}}-{\text{t}}_{0}))){\;(\text{I}-\text{exp}\;({\text{A}}^{\text{c}}\text{T}))}^{-1}B_{\text{c}}{\text{U}}_{\text{basal}}$$

Notice that this formula can be generalized for patients with two basal dose injections a day by defining the basal doses time as ${\text{t}}_{{\text{B}}_{1}}$ and ${\text{t}}_{{\text{B}}_{2}}$ , and the basal doses as ${\text{U}}_{\text{basa}{\text{l}}_{1}}$ and ${\text{U}}_{\text{basa}{\text{l}}_{2}}$ .

### A.3 Table of Symbols

**Table:**

Symbol	Description	Units
*G*	Subcutaneous glucose as measured by CGM	mg/dL
*G_basal_*	Capillary blood glucose as measured by glucose meter	mg/dL
*G_b_*	Model parameter for basal glucose	mg/dL
*G_t_*	Glucose target	mg/dL
*TDI*	Total daily insulin	units
*U_basal_*	Injected basal insulin dose	units
*B_c_*	Injected basal insulin dose at day c	units
$B_{C}^{opt}$	Optimized basal insulin dose to be given at day c	units
*U_bolus_*	Injected bolus insulin dose	units
*U_meal_*	Counted carbohydrate content of consumed meal	g
*t_B_*	Time of injected basal insulin	min
*T*	Period of injecting basal insulin	min
*f^m^*	Model parameter for bioavailability of meal m	_
$k_{q1}^{m},k_{q2}^{m},k_{q12}^{m}$	Model parameters for time constants of meal absorption	min
$S_{i}^{d}$	Model parameter for insulin sensitivity of day d	1/min per mU/L
